# There Is No Substitute for Effective Education About Biosimilars

**DOI:** 10.1093/crocol/otab047

**Published:** 2021-07-07

**Authors:** Ross M Maltz, Megan McNicol, Laura Wingate, Sarah Buchanan, Angela Sandell, Sandra C Kim, David T Rubin

**Affiliations:** 1 Division of Pediatric Gastroenterology, Hepatology, and Nutrition, Nationwide Children’s Hospital, Columbus, Ohio, USA; 2 Department of Pediatrics, The Ohio State University Wexner Medical Center, Columbus, Ohio, USA; 3 Department of Pharmacy, Nationwide Children’s Hospital, Columbus, Ohio, USA; 4 Crohn’s & Colitis Foundation, New York, New York, USA; 5 Division of Gastroenterology, Hepatology, and Nutrition, Department of Pediatrics, Golisano Children’s Hospital, Rochester, New York, USA; 6 Division of Gastroenterology, Hepatology, and Nutrition, Department of Pediatrics, University of Pittsburgh Medical Center, Pittsburgh, Pennsylvania, USA; 7 University of Chicago Medicine Inflammatory Bowel Disease Center, Chicago, Illinois, USA

Biologic agents, which include tumor necrosis factor (anti-TNFs) alpha inhibitors, are highly effective at inducing and maintaining remission, achieving mucosal healing, and reducing hospitalizations and surgeries in patients with inflammatory bowel disease (IBD).^1–3^ However, biologic agents are also the largest driver of health care expenditures for patients with IBD,^4^ with an estimated 50% increase in biologic spending from 2014 to 2018.^5^ In an effort to decrease health care costs and drive market competition, the Biologics Price Competition and Innovation Act was passed in 2009, which created an abbreviated approval pathway for biosimilars. A biosimilar is a biological product that is highly similar to and has no clinically meaningful differences from an existing Food and Drug Administration (FDA) approved reference product.^6^ Approval is granted when an equivalency study is performed for at least one indication of the reference (originator) drug and is then granted extrapolation of the label to all indications for that drug. The first anti-TNF biosimilar was approved by the European Medicines Agency’s Committee in June 2013 and by the US FDA in 2016. There are now 4 FDA-approved biosimilars to infliximab and 6 FDA-approved biosimilars to adalimumab. European countries have reported significant cost savings through the utilization of biosimilars both in naïve patients and in patients switching from the originator to a biosimilar.^7, 8^ The increased utilization of biosimilars in the United States has also resulted in significant cost savings.^9–11^ Despite the cost of biosimilars in the United States being 10%–50% less than the originator drug,^12^ biosimilars currently account for only 0.9% market share.^13^ Identifiable barriers to biosimilar adoption include a lack of provider and patient education on biosimilars and subsequently, little change in prescribing habits. Additionally, there are ongoing pharmaceutical rebate agreements between payors and pharmaceutical companies that lessen the financial incentives to utilize a biosimilar product.^14^

Biosimilars are proven to have similar efficacy, immunogenicity, and safety to the originator,^15–18^ and their use in IBD treatment is supported by the European Crohn’s Colitis Organisation^19^ and the Crohn’s & Colitis Foundation.^20^ The use of anti-TNF biosimilars in anti-TNF naïve patients has been comparable in adult patients with IBD, demonstrating no significant difference in primary nonresponse, remission rate, secondary loss of response, or adverse events in patients initiating a biosimilar compared with the originator.^17, 18^ Studies in pediatric patients with IBD have also demonstrated similar efficacy, immunogenicity, and safety with biosimilar use, although the data are more limited in the pediatric population.^11, 21^

Importantly, switching from the originator to a biosimilar has also shown similar efficacy, immunogenicity, safety, and quality of life in both adult and pediatric patients with IBD.^14, 17, ^^21–24^ While the safety, efficacy, and cost savings of switching biologic products have been established, it is also recognized that this process requires a collaborative effort from health care providers, pharmacists, nurses, payors, institutions, and patients.^14, 25^

Despite these data, there is a great deal of hesitancy by providers and patients to utilize biosimilars in the care of patients with IBD. This may in part be due to a lack of patient and provider education and familiarity with biosimilars. Despite the medical literature demonstrating the safety and efficacy of biosimilars,^15–18^ 49% of IBD providers were not comfortable with its use.^26^ Pediatric IBD providers were even less comfortable recommending a biosimilar compared to adult IBD providers.^26^ This reluctance may stem from provider hesitancy regarding how to explain biosimilars to their patients^19, 27^ or a fear of patient resistance due to unfamiliarity with these products.^28–31^ Patient awareness and understanding is vital and is an essential factor impacting acceptance of treatment with a biosimilar agent.^32^ Recently, Morris et al demonstrated the successful use of quality improvement methodology to increase biosimilar use from 1% to 96% in intravenous (IV) anti-TNF naïve pediatric patients. This was accomplished through extensive provider and patient education as well as streamlining the prior authorization process for all inpatient and outpatient biosimilar initiations.^11^ In Europe, provider acceptance towards biosimilars changed with increased knowledge and experience.^33^

The importance of proper patient and provider education is even more critical when considering a switch from the originator to a biosimilar since 74% of patients with IBD have not heard of biosimilars.^34^ There is therefore an understandable reluctance and negative association towards switching among patients, and this hesitancy can adversely impact patient outcomes; this is commonly referred to as the “nocebo effect”. ^14, 23, 32, 35, 36^ Provider buy-in, clear communication, and patient reassurance are associated with a reduced nocebo effect and are critical to overcome the barriers to successful switching in both pediatric and adult patients with IBD.^37, 38^

Furthermore, there should be appropriate safeguards and acceptable exclusions for some situations involving biosimilar switching. Single switches should be only considered in patients in clinical remission, on stable dosing, and with therapeutic drug levels. Health care providers should have the ability to monitor clinical and mucosal outcomes before and after such switches occur. Such disease and drug monitoring will improve the confidence of both patient and provider prior to switches, and arguably improve care with the opportunity to optimize treatment. The recent formulary changes from payors have made shared decision making between patients and providers difficult since the insurance mandate to switch a patient from the originator to a biosimilar often comes with insufficient notification to allow for education, reassurance, and discussion.^39^ Importantly, at the current time, multiple switches between the originator and biosimilars are not recommended^20^ due to a lack of sufficient data supporting the safety and efficacy in patients with IBD.

Increased use of biosimilars presents an opportunity to significantly reduce health care costs and improve patient access to biologic therapies by increasing market share to create competition and decreasing overall treatment costs, while maintaining a high level of patient care. However, this is only possible with appropriate education and altered prescribing habits of health care providers. Additionally, patient-facing education is vital to achieve buy-in and reduce the nocebo effect. The application of effective quality improvement strategies, ongoing research, and continued advocacy and partnership with insurance providers, pharmaceutical companies, and legislatures is vital to move this forward. By improving provider and patient knowledge of biosimilars and their use, there will be a positive impact for patients and the health care system in general by decreasing cost of IBD care, improving medication access, and impacting long-term clinical and quality of life outcomes for patients living with IBD. Effective collaboration between payors, providers, and patients is vital for increased biosimilar use. Payors must provide adequate notification, education, time for discussion and reassurance, and support appropriate disease and drug monitoring. The return on these investments will be greater biosimilar uptake, incremental cost savings, and improved patient care.

## Conflict of Interest

R.M.M., M.M., S.B., A.S., S.C.K. have no conflicts of interests. L.W. is a member of the American Board of Internal Medicine Gastroenterology Biosimilar SteerCo. D.T.R. has received grant support from Takeda; and has served as a consultant for Abbvie, Altrubio, Allergan Inc., Arena Pharmaceuticals, Bellatrix Pharmaceuticals, Boehringer Ingelheim Ltd., Bristol-Myers Squibb, Celgene Corp/Syneos, Connect BioPharma, GalenPharma/Atlantica, Genentech/Roche, Gilead Sciences, InDex Pharmaceuticals, Ironwood Pharmaceuticals, Iterative Scopes, Janssen Pharmaceuticals, Lilly, Materia Prima, Pfizer, Prometheus Biosciences, Reistone, Takeda, and Techlab Inc.
